# Association of elevated levels of peripheral complement components with cortical thinning and impaired logical memory in drug-naïve patients with first-episode schizophrenia

**DOI:** 10.1038/s41537-023-00409-1

**Published:** 2023-11-07

**Authors:** Hua Yu, Peiyan Ni, Yang Tian, Liansheng Zhao, Mingli Li, Xiaojing Li, Wei Wei, Jinxue Wei, Qiang Wang, Wanjun Guo, Wei Deng, Xiaohong Ma, Jeremy Coid, Tao Li

**Affiliations:** 1 Affiliated Mental Health Center & Hangzhou Seventh People’s Hospital and School of Brain Science and Brain Medicine, Zhejiang University School of Medicine, Hangzhou, 310058 China; 2 Liangzhu Laboratory, MOE Frontier Science Center for Brain Science and Brain-machine Integration, State Key Laboratory of Brain-machine Intelligence, Zhejiang University, Hangzhou, 311121 China; 3 NHC and CAMS Key Laboratory of Medical Neurobiology, Zhejiang University, Hangzhou, 310058 China; 4 Psychiatric Laboratory and Mental Health Center, West China Hospital, Sichuan University, Chengdu, 610041 China

**Keywords:** Schizophrenia, Schizophrenia

## Abstract

Schizophrenia has been linked to polymorphism in genes encoding components of the complement system, and hyperactive complement activity has been linked to immune dysfunction in schizophrenia patients. Whether and how specific complement components influence brain structure and cognition in the disease is unclear. Here we compared 52 drug-naïve patients with first-episode schizophrenia and 52 healthy controls in terms of levels of peripheral complement factors, cortical thickness (CT), logical memory and psychotic symptoms. We also explored the relationship between complement factors with CT, cognition and psychotic symptoms. Patients showed significantly higher levels of C1q, C4, factor B, factor H, and properdin in plasma. Among patients, higher levels of C3 in plasma were associated with worse memory recall, while higher levels of C4, factor B and factor H were associated with thinner sensory cortex. These findings link dysregulation of specific complement components to abnormal brain structure and cognition in schizophrenia.

## Background

Schizophrenia is a severe psychiatric disorder that affects approximately 1% of the global population^1^, impairing their social function and shortening life expectancy^2^. The disease appears to have genetic and environmental causes^3–6^. Polymorphism in the gene encoding C4 in the complement system has been linked to risk of schizophrenia^7^, and excessive complement activity has been linked to immune dysfunction that may contribute to the disease^8^. In addition, a single-nucleotide polymorphism in the gene encoding CSMD1, which degrades the complement components C3 and C4^9^, has been strongly associated with risk of the disease^10^.

Post-mortem analysis of brains from individuals with schizophrenia has revealed increased C4 expression in hippocampal subfields, para-hippocampal gyrus, insula entorhinal, medial orbitofrontal cortex, and posterior cingulate^7,11^. Consistently, the same regions show cortical thinning in studies of brains from living individuals with schizophrenia^7,11–13^. In addition, C4 overexpression in mice reduces the density of cortical synapses, increases microglial engulfment of synapses, and induces behavioral deficits^14^. The complement system as a whole promotes synaptic pruning^11^ and neuronal migration during development^15^, so it is tempting to speculate that hyperactive complement activity may lead to excessive synaptic pruning and other neuropathology that contribute to schizophrenia^11,16^.

Consistent with this hypothesis, C4 overexpression has been linked to worse memory in individuals with schizophrenia as well as in healthy controls^17^, and with more severe delusional symptoms in the disease^18^. C3 overexpression has been linked to memory loss and brain volume reduction in multiple sclerosis^19^, and polymorphism in genes encoding complement components other than C4 has been linked to worse memory in individuals with psychosis as well as in healthy controls^20^.

While substantial evidence links overexpression of C4 and C3 to greater risk or severity of schizophrenia and associated neuropathology, some studies have failed to detect elevated levels of either complement component in the plasma of affected individuals^21–23^. In addition, few studies have examined whether levels of other complement components are elevated in affected individuals^24^, which is an important question to address given that complement responses can proceed not only via the “classical” and “lectin” pathways involving C4^25^, but also *via* the “alternative” pathway involving factor B, factor H and properdin^26,27^.

Here we systematically compared the levels of numerous key complement components in plasma between individuals with schizophrenia and healthy controls. We recruited only drug-naïve individuals who had experienced their first schizophrenic episode, allowing us to exclude potential confounding due to antipsychotic drug use and episode of disease^28^. We also explored potential associations of complement levels with memory, cortical thickness, and psychotic symptoms.

## Methods

### Participants

The study procedure was approved by the Ethics Committee of West China Hospital, Sichuan University. All participants were recruited between March 2014 and February 2019 from in- and outpatient psychiatric facilities at West China Hospital of Sichuan University. Individuals with schizophrenia were enrolled if they had experienced their first episode as determined by two experienced psychiatrists based on the fourth edition of the *Diagnostic and Statistical Manual of Mental Disorders* and the Structured Clinical Interview for DSM-IV Disorders^29^. Symptom severity was measured using the Positive and Negative Symptom Rating Scale^30^. We excluded individuals who had ever taken antipsychotic drugs for more than three days, met the criteria for alcohol or substance abuse within one year prior to their screening for this study, reported a history of any significant medical illness, or had contraindications to magnetic resonance imaging (MRI). Healthy controls were screened for major psychiatric disorders using the “non-patient” version of the Structured Clinical Interview for DSM-IV Disorders. None reported being on psychotropic medication. All participants were right-handed, and all provided written informed consent before enrollment.

### Logical memory recall

Immediate and delayed logical memory recall were assessed using the Chinese version of the short form of the revised Wechsler Adult Intelligence Scale^31^ containing the following seven subtests: information, arithmetic, digital symbol, digital span test, block design, picture completion, and similarities. Scores for immediate and delayed logical memory recall ranged from 0 to 25, with higher scores indicating greater recall^32^. The intelligence quotient (IQ), verbal IQ (VIQ) and performance IQ (PIQ) were also measured. The Supplementary Methods contain additional details about cognitive assessment.

### Cortical thickness by MRI

All participants underwent MRI scanning on a 3.0-T Achieva system (Philips, Amsterdam, The Netherlands) equipped with an eight-channel phased-array head coil. To minimize head movement and scanner noise, foam padding and earplugs were used. High-resolution T1 images were acquired using a 3D magnetization-prepared rapid gradient-echo sequence with the following parameters: repetition time, 8.37 ms; echo time, 3.88 ms; flip angle, 7°; in-plane matrix resolution, 256 × 256; field of view, 24 × 24 cm^2^; and number of slices, 188. T1-weighted MRI scans from all participants were processed using the standardized pipeline of CAT12 and smoothed using a Gaussian kernel with full-width at half-maximum of 12 mm, generating brain maps of cortical thickness.

### Levels of complement components in plasma

The concentrations of complement components in plasma were assayed as described^33^. Briefly, blood samples were collected *via* venipuncture at 4.00–4.30 p.m. into tubes containing ethylenediaminetetraacetic acid. Blood was centrifuged at 1000 *g* for 10 min at 4 °C to remove peripheral blood mononuclear cells, and the plasma was immediately divided into 0.5-mL aliquots and stored at −80 °C. Plasma factors were assayed using MILLIPLEX^®^ MAP kits (Merck, Darmstadt, Germany), while the complement components C1q, C3, C3b/iC3b, C4, factor B, properdin, and factor H were assayed using the Human Complement Panel 2-Immunology Multiplex Assay (catalog no. HCMP2MAG-19K, Merck Millipore, Billerica, MA, USA). Kits were used according to the manufacturer’s instructions, and data were analyzed using a FLEXMAP 3D^®^ instrument (Luminex, Merck Millipore) and xPONENT^®^ 4.0 software (Luminex). Standard curves were generated using the standards in the kit. Median fluorescence intensity was converted into concentrations using a weighted 5-parameter logistic method. Supplementary Figs. 1–2 show additional details of complement assay in plasma.

### Statistical analysis

All analyses were performed using SPSS 24.0 (SPSS, Chicago, IL, USA) and STATA 14.0 (Stata Corporation, College Station, Texas, USA). Differences between individuals with schizophrenia and healthy controls in continuous demographic variables were assessed for significance using a two-sample *t* test, while differences in categorical variables were assessed using a chi-squared test. Normal distribution of all variables was verified using the Shapiro-Wilk test. Scores for immediate or delayed logic memory recall were compared between individuals with schizophrenia and healthy controls using analysis of covariance that took into account age, sex, and education level based on Bonferroni correction. Concentrations of complement components were log_10_-transformed to normalize their distributions, then compared between individuals with schizophrenia and healthy controls while controlling for age, sex, and body mass index.

Vertex-wise cortical thickness was compared between individuals with schizophrenia and healthy controls using a two-sample *t* test in which we controlled for age, sex, education level and total intracranial volume. Potential associations of cortical thickness with levels of complement components were explored separately among individuals with schizophrenia, among healthy controls, and among all participants in regression that controlled for age, sex, education level, body mass index and total intracranial volume^34,35^. Stepwise multiple linear regressions were performed between logical memory recall and complement components among individuals with schizophrenia, among healthy controls, and among all participants. Before regression, we used the “least absolute shrinkage and selection operator” (LASSO) in STATA^36^ to select the complement factors that were associated with logical memory recall in order to avoid multicollinearity: levels of complement components correlated strongly with one another, with correlation coefficients >0.7 (Supplementary Table 1). After LASSO selection, immediate logical memory recall across all participants was analyzed using stepwise multiple linear regression in which the independent variables were age, sex, education, body mass index, and log_10_-transformed concentrations of C4 and factor H. Delayed logical memory recall across all individuals with schizophrenia was analyzed in stepwise multiple linear regression in which the independent variables were age, education, body mass index, and log_10_-transformed concentrations of C1q, C3, C4, and properdin. Insignificant variables were removed sequentially until only significant ones remained. Potential associations of cortical thickness with logical memory recall were explored to determine whether brain cortical thickness associated with complement factors could potentially mediate the relationship between plasma complement components and logical memory; this analysis controlled for age, sex, education level, and body mass index. In addition, partial correlation analysis was used to evaluate the correlation between plasma complement measurements and clinical parameters after controlling for age, sex, education level, and body mass index. Significance was defined as *p* < 0.05. During analysis of MRI images, significance was defined as vertex-wise *p* < 0.001 after correction for family-wise error rate at the cluster level.

## Results

**Fig. 1:**
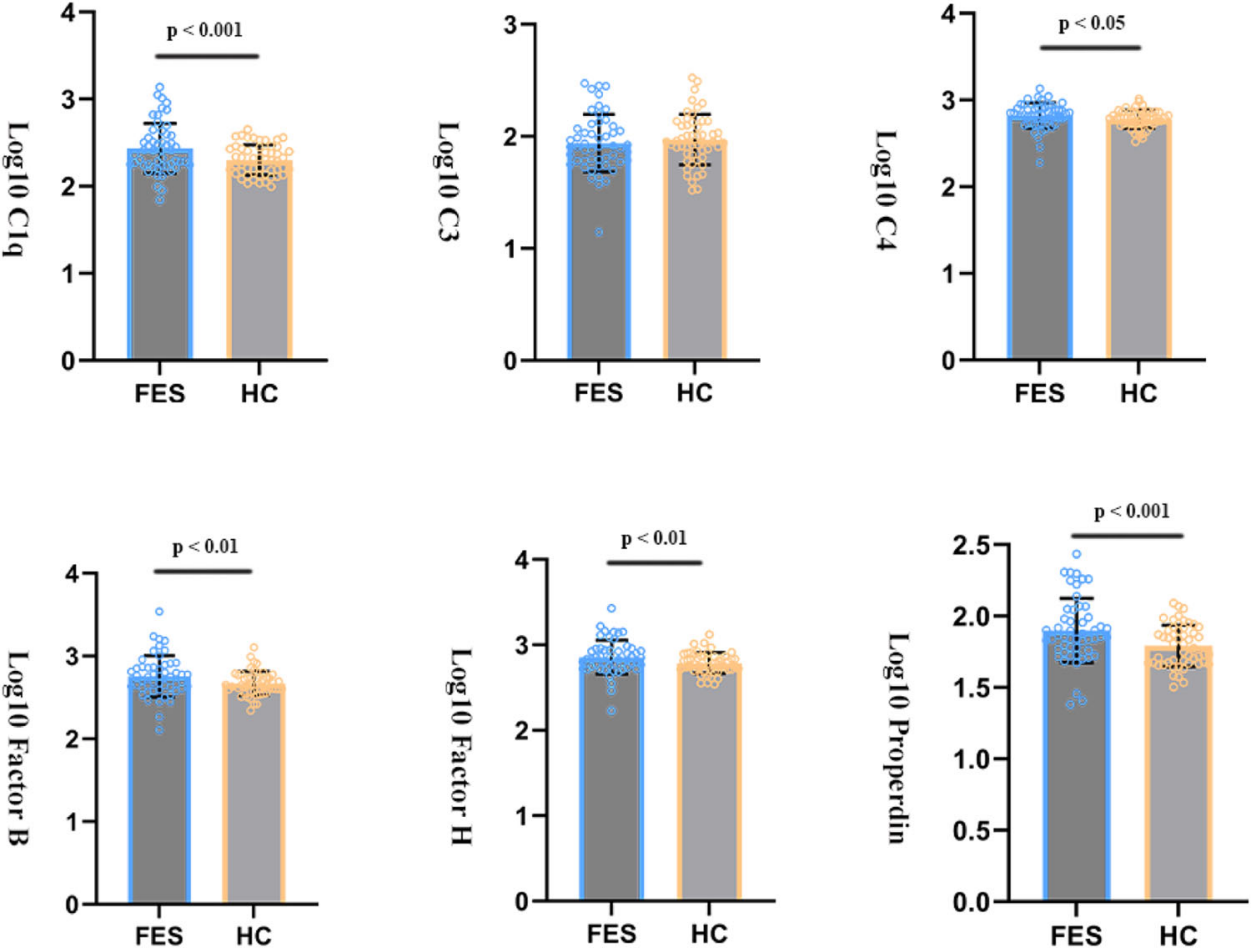
Complement protein concentrations in patients and controls. Peripheral protein concentrations of complement components show significant increases in C1q, C4, factor B, factor H, and properdin in first-episode schizophrenia (FES) patients compared to controls. No difference was observed for C3. Bars represent the mean ± SD, and p-values are adjusted for age, sex, and body mass index (BMI). Abbreviations: FES: first episode schizophrenia; HC: healthy control; SD: standard deviation.

We screened 62 individuals with schizophrenia and 54 healthy controls who visited our in- or outpatient facilities during the enrollment period. In the end, we enrolled 52 drug-naïve individuals with first-episode schizophrenia (24 men) and 52 healthy controls (21 men) (Supplementary Fig. 3). Individuals with schizophrenia were younger than healthy controls and obtained lower verbal, performance and total IQ scores^37^. They also showed significantly worse immediate and delayed logical memory recall than controls, and they showed significantly higher levels of C1q, C4, factor B, factor H and properdin in plasma, but similar levels of C3 as controls (Table 1, Fig. 1).

**Table 1:** Demographic and clinical characteristics of FES and HC.

	FES M (SD) (*n* = 52)	HC M (SD) (*n* = 52)	*F/t / x²*	df	*P*, 2-tail	Post hoc test
Age (Years)	21.71 (7.16)	27.65 (8.97)	−3.74	102	0.000***	FES < HC
Education (years)	11.24 (2.97)	15.22 (3.62)	−6.13	102	0.000***	FES < HC
BMI	20.49 (3.19)	21.34 (3.23)	−1.35	102	0.18	-
Gender (male /female)	24 (M)/ 28 (F)	21 (M)/ 31 (F)	0.35	1, 104	0.55	-
Smoking (yes/no)	7 (yes)/33(no)	5 (yes)/44 (no)	1.01	1, 89	0.32	-
*ANCOVA*						
VIQ	98.70 (17.32)	112.41 (13.57)	8.64	1, 94	0.004**	FES < HC
PIQ	91.28 (18.30)	108.49 (13.13)	16.91	1, 94	0.000***	FES < HC
WAIS IQ	95.07 (15.87)	111.67 (13.47)	16.577	1, 94	0.000***	FES < HC
Logical memory immediately	7.30 (4.65)	10.86 (4.26)	10.65	1, 95	0.002**	FES < HC
Logical memory delayed	5.74 (3.95)	8.67 (4.35)	5.54	1, 94	0.021*	FES < HC
TMT-A-Time	40.07 (15.03)	37.25 (14.70)	1.24	1, 95	0.27	-
TMT-B-Time	69.41 (30.45)	56.13 (25.55)	2.99	1, 95	0.087	-
DSST	42.29 (14.20)	59.61 (19.90)	11.55	1, 93	0.001**	FES < HC
Log_10_ C1q	2.44 (0.29)	2.30 (0.17)	13.05	1, 104	0.000***	FES > HC
Log_10_ C3	1.94 (0.26)	1.97 (0.23)	0.045	1, 104	0.083	-
Log_10_ C4	2.82 (0.15)	2.78 (0.10)	6.50	1, 104	0.012*	FES > HC
Log_10_ Factor B	2.75 (0.25)	2.67 (0.14)	7.21	1, 104	0.008**	FES > HC
Log_10_ Factor H	2.86 (0.20)	2.79 (0.12)	9.99	1, 104	0.002**	FES > HC
Log_10_ Properdin	1.90 (0.23)	1.79 (0.15)	14.63	1, 104	0.000***	FES > HC
*Descriptive Analysis*						
PANSS total score	82.16 (22.90)	-	-	-	**-**	-
PANSS PS score	21.43 (6.48)	-	-	-	**-**	-
PANSS NS score	22.04 (8.59)	-	-	-	**-**	-
PANSS GP score	38.69 (11.34)	-	-	-	-	-
GAF	49.36 (16.68)	-	-	-	-	-
Illness duration (months)	13.05 (21.63)	-	-	-	-	-

For the comparison of complement factors, age, gender, and BMI were co-variated out.

*FES* first episode schizophrenia, *HC* healthy controls, *WAIS* Wechsler adult intelligence scale vocabulary score (37 items), *IQ* intelligence quotient, *VIQ* verbal intelligence quotient, *PIQ* performance intelligence quotient, *TMT-A* trail making test parts A, *TMT-B* trail making test parts B, *DSST* digit symbol substitution test, *C1q* complement component 1q, *C3* complement component 3, *C4* complement component 4, *Factor B* complement factor B, *Factor H* complement factor B, *PANSS* the positive and negative symptom scale, *PS* positive symptom scale, *NS* negative symptom scale, *GP* general psychopathology, *GAF* global assessment of functioning, *n* number, *SD* standard variance, *M* male, *F* female. For the comparison of cognitive function.

*p < 0.05, **p < 0.01, ***p < 0.001. Bold represents statistically significant results (*p* < 0.05).

### Associations between levels of complement components and memory recall

We examined potential associations between levels of complement factors and immediate logical memory scores across all study participants. In Step 1, education level significantly explained the variance in scores (*F* = 10.86, *p* < 0.01; *R*^2^ = 0.10; β = 0.39), which did not change in Step 2 after inclusion of C4 concentration (*F* = 8.10, *p* < 0.05; *R*^2^ = 0.15; β = −7.94; Table 2, Supplementary Table 2). Our results indicate that higher levels of C4 were associated with worse scores for immediate logical memory recall.

**Table 2:** Association between complement factors and immediate logical memory recall function in the combined groups.

	Unstandardized Coefficients		Standardized Coefficients	*t*	*p*	*R ²*	Adjusted *R ²*	*F*
	*B*	SE	*Beta*					
*Model 1*								
Constant	3.93	1.67	-	2.35	<0.05	0.10	0.094	*F* (1, 93) = 10.86, *p* < 0.01
Education years	0.39	0.12	0.32	3.29	<0.01			
*Model 2*								
Constant	26.21	10.16	-		<0.05	0.15	0.13	*F* (2, 92) = 8.10, *p* < 0.01
Education years	0.39	0.12	0.32	3.31	<0.01			
Log_10_ C4	−7.94	3.58	−0.21	−2.22	<0.05			

Independent variable in the regression model after least absolute shrinkage and selection operator (LASSO) selection: age, gender, education, log10 C4, and log10 Factor H.

*SE* standardized error.

Similarly, we examined potential associations between levels of complement factors and delayed logical memory scores across individuals with schizophrenia but not in healthy controls. We found that higher C3 concentration was associated with worse scores for delayed logical memory recall (Table 3, Supplementary Table 2).

**Table 3:** Association between complement factors and delayed logical memory recall function in FES group.

	Unstandardized Coefficients		Standardized Coefficients	*t*	*p*	*R* ²	Adjusted *R* ²	*F*
	*B*	SE	*Beta*					
*Model 1*								
Constant	14.97	4.57	-	3.28	<0.005	0.092	0.070	*F* (1, 41) = 4.15, *p* < 0.05
Log_10_ C3	−4.76	2.34	−0.30	−2.04	<0.05			

Independent variable in the regression model after least absolute shrinkage and selection operator (LASSO) selection: age, education, BMI, log_10_ C1q, log_10_ C3, log_10_ C4, and log_10_ properdin.

*SE* standardized error, *FES* first episode schizophrenia.

### Associations between levels of complement components and cortical thickness

**Fig. 2:**
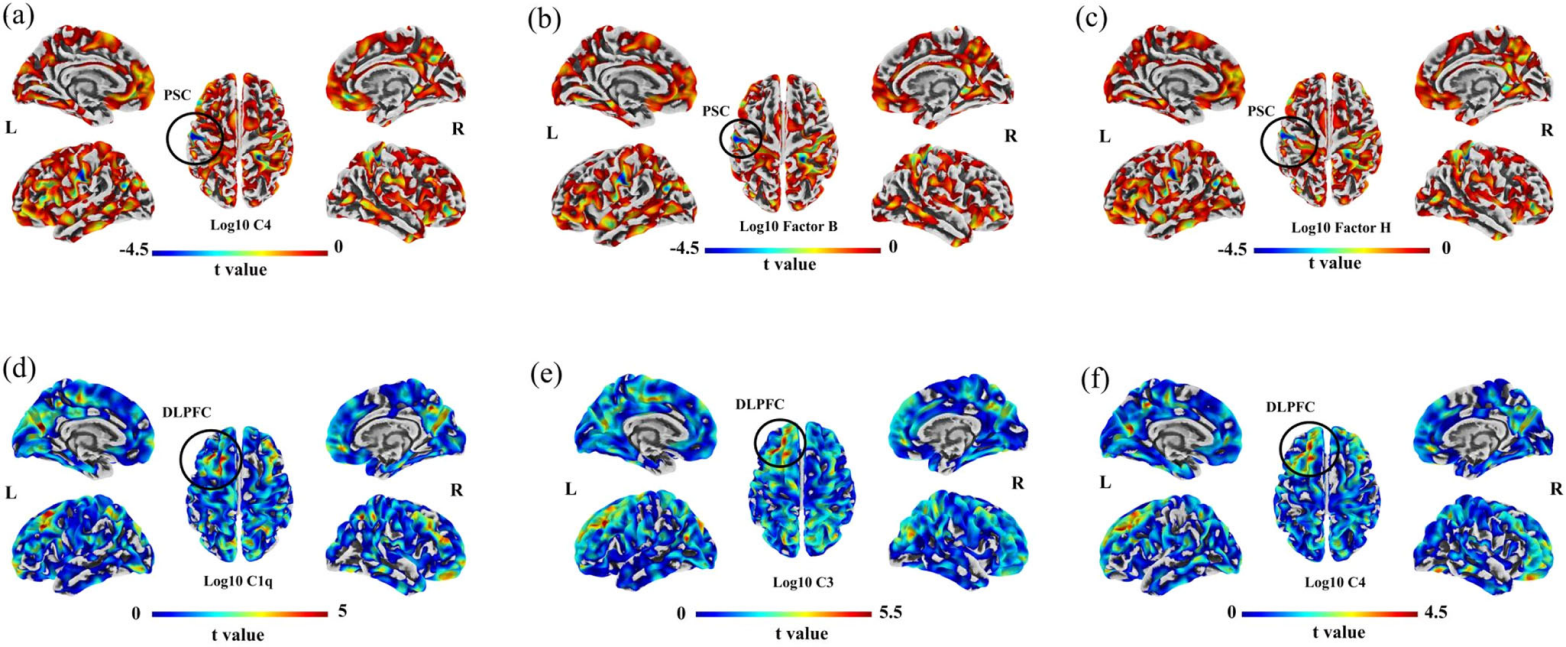
Linear regression between complement components and cortical thickness in FES and healthy controls. **a**–**c** Linear regression analyses (adjusting for age, sex, education years, BMI and TIV) in FES revealed a significant negative correlation between the complement component 4, factor B and factor H with individual frontal thickness regions that made up the left primary sensorimotor cortex (PSC). The peak Montreal Neurological Institute (MNI) coordinates of left PSC associated with C4 is *X* = −57/−60, *Y* = −18/−14, *Z* = 32/39, *t* = −4.02/−4.01, cluster size = 121, corrected *p* < 0.01; factor B is *X* = −57/−60, *Y* = −18/−14, *Z* = 31/39, *t* = −4.14/−3.98, cluster size = 103, corrected *p* < 0.01; factor H is *X* = −57/−60, *Y* = −18/−14, *Z* = 32/39, *t* = −4.21/−3.87, cluster size = 103, corrected *p* < 0.01. Blue-colored regions (in dark circle) show areas with a significantly negative correlation of CT with complement factors in participants with FES (cluster *p* < 0.05, FWE corrected; vertex level *p*_uncorrected_ < 0.001). No significant positive correlations were detected in FES patients. **d**–**f** Linear regression analyses (adjusting for age, gender, education years, BMI and TIV) in healthy controls revealed a significant positive correlation between the plasma complement C1q, complement component 4, and complement component 3 with the left dorsal lateral prefrontal cortex (DLPFC). The peak MNI coordinates of left DLPFC associated withC1q is *X* = −23/−21, *Y* = 27/10, *Z* = 54/50, *t* = 4.42/4.34, cluster size = 132, corrected *p* < 0.01; C3 is *X* = −34/−22/−20, *Y* = 31/43/29, *Z* = 38/34/40, *t* = 5.31/4.69/3.82, corrected *p* < 0.001; C4 is *X* = −20/−23/−20,*Y* = 41/17/30, *Z* = 34/43/38, *t* = −4.01/3.56/3.42, cluster size = 100, corrected *p* < 0.05. Red-colored region (in the white circle) shows the area with a significantly positive correlation of CT with complement factors in control participants (cluster *p* < 0.05, FWE corrected; vertex level *p*_uncorrected_ < 0.001). No significant negative correlations were detected in the controls. Abbreviations: FES first episode schizophrenia, BMI body mass index, TIV total intracranial volume, PSC primary sensory cortex, CT cortical thickness, DLPFC dorsolateral prefrontal cortex, FWE family wise error.

Among individuals with schizophrenia, thickness of the left primary sensory cortex was inversely associated with levels of C4, factor B, and factor H after controlling for age, sex, education level, body mass index, and total intracranial volume (Fig. 2a–c). Among healthy controls, thickness of the left dorsolateral prefrontal cortex was positively associated with levels of C1q, C3 and C4 after controlling for age, sex, education level, body mass index, and total intracranial volume (Fig. 2d–f).

When we analyzed individuals with schizophrenia and healthy controls together, we did not detect any significant relationships between the level of any complement component and thickness of any cortical brain region. We did not observe any significant association between cortical thickness and either immediate or delayed logical memory recall among individuals with schizophrenia, among healthy controls or among all subjects together after controlling for age, sex, education level, body mass index, and total intracranial volume. Our results suggest that variations in cortical thickness in our sample did not contribute to the association between levels of complement factors and logical memory recall.

### Associations between levels of complement components and psychotic symptoms

Among individuals with schizophrenia, we did not observe any significant correlations between levels of complement components and illness duration or total score or sub-scale scores on the Positive and Negative Symptom Rating Scale after controlling for age, sex, education level and body mass index (Supplementary Table 3).

## Discussion

Our controlled analysis of drug-naïve individuals with first-episode schizophrenia suggests hyperactivation of all three complement cascades in the disease and links such hyperactivation to thinning of the primary sensory cortex in affected individuals. It also links such hyperactivation to impairment of logical memory in affected and healthy individuals, consistent with a study involving individuals with psychosis and healthy controls^17^. In the present work, levels of different complement components showed different relationships to cortical thickness in the brain depending on whether schizophrenia was present or not: in affected individuals, higher levels of C4, factor B and factor H were associated with thinner primary sensory cortex; in healthy controls, higher levels of C1q, C4 and factor H were associated with thicker left dorsolateral prefrontal cortex. To our knowledge, this is the first report linking the complement cascade to abnormalities in the primary sensorimotor network.

Our findings of elevated levels of complement components in drug-naïve, first-episode schizophrenia are consistent with previous studies reporting upregulation of C4^7,11,14^ and factor B^38^ in individuals with multi-episode schizophrenia, upregulation of C1q in individuals with first-episode schizophrenia^39^, increased activity of C1 in patients with multiple-episode schizophrenic in the acute stage of the disease^40^, and upregulation of factor H in the hippocampus of individuals with stable schizophrenia who had been ill for fewer than five years^41^. Thus, substantial evidence points to hyperactivation of the classical, lectin and alternative complement pathways in schizophrenia. This is a key contribution of our study given that components of the alternative pathway, such as factor B, factor H and properdin, have not been extensively explored in schizophrenia.

Our findings contrast with studies reporting normal activation of the lectin complement pathway in multiple-episode, drug-treated schizophrenia^42^ or normal levels of properdin in individuals with first-episode psychosis^28^. We consider that our results may be more reliable because we eliminated confounding due to medication, disease episode and factors known to influence complement levels such as body mass index and smoking status^43^. Our work suggests that previously reported associations between complement hyperactivation and multi-episode schizophrenia also apply to drug-naïve, first-episode disease.

Our work also strengthens the idea that complement hyperactivation contributes to immune dysregulation in the condition^11^. Indeed, the genomic region most strongly associated with schizophrenia risk contains genes of the major histocompatibility complex as well as the gene encoding C4^44^, and this genomic region has been implicated in various autoimmune diseases^11^. Prenatal exposure to maternal immune activation in mice has been linked to disruption of cytoarchitecture in the primary somatosensory cortex^45^; such disruption, together with brain activation, may help explain the abnormal behavior of mouse models of autism and other neurodevelopmental disorders. The disrupted cortical cytoarchitecture in that previous work is reminiscent of our present finding of cortical thinning in the primary sensory cortex of individuals with schizophrenia.

Despite the strong evidence for hyperactivation of the classical complement pathway in schizophrenia, levels of C3 were not significantly higher in our individuals with schizophrenia than in healthy controls, which is consistent with a meta-analysis comparing individuals with schizophrenia and controls^24^ and with a study of individuals with first-episode psychosis^43^. In contrast, another study reported higher levels of C3 in chronic schizophrenia patients than in healthy controls^11,46^. One potential explanation for these discrepant reports is that C3 is not related to risk of schizophrenia in Han Chinese, among whom individuals with schizophrenia and healthy controls do not appear to differ significantly in frequencies of polymorphisms in the gene encoding C3 or in plasma levels of C3^47^.

The aforementioned study of individuals with first-episode psychosis^28,43^ found no significant abnormality in levels of C1q or C4, both of which were elevated in our participants with schizophrenia. This difference may reflect that many subjects in the psychosis study did not have schizophrenia or were taking medication, and we cannot exclude the influence of differences in smoking status, ethnicity or body mass index^43^. Our analysis links upregulation of C3 and C4 to worse logical memory recall not only in individuals with first-episode schizophrenia but also in healthy controls, consistent with studies involving healthy individuals^17,48^ or those with multiple sclerosis or stroke^17,19,49^. A possible explanation for these findings is that complement proteins, especially C4, aid in synaptic pruning^14,50^ and that excessive pruning can lead to synaptic loss like that observed in schizophrenia^51^. C3 hyperactivity in a mouse model of autoimmune encephalomyelitis has been linked to early dendritic loss and microglia-mediated phagocytosis of synapses in the hippocampus, and inhibiting C3 in these animals mitigates memory impairment^19^. Our work supports the idea that inhibiting complement pathways may ameliorate memory deficits in schizophrenia^17^ and other neurological disorders^52,53^.

On the other hand, our work does not shed light on whether and how the logical memory defect in our individuals with schizophrenia may be related to their cortical thinning. Similarly, a study of individuals with psychosis failed to find an association between cognitive function (including digital number memory) and brain cortical thickness^54^. Further work is needed to clarify whether and how complement levels influence brain structure, and whether and how the altered structure affects cognitive function. Assuming that the elevated complement levels in our individuals with schizophrenia contribute to the observed cortical thinning, we suggest that complement hyperactivation is a chronic, not acute, feature of the disease, given that neuroanatomical changes take time to appear^11^.

Our work failed to find significant associations between levels of complement components and severity of schizophrenia symptoms measured on the Positive and Negative Symptom Rating Scale. A previous study also found no significant association of levels of C3 or C4 with scores on the same scale among individuals with multiple-episode, drug-treated schizophrenia^55,56^, while an analysis of 7000 children found no association between C4 expression and occurrence of psychotic experiences during childhood^57^. These negative findings highlight the complexity of elucidating whether and how the complement system contributes to the clinical manifestations of schizophrenia. Given that levels of interleukins 1β, 6 and 8 as well as tumor necrosis factor-α have been linked to psychotic symptoms in schizophrenia^58^, we recommend that future research explore whether and how not only complement components but also inflammatory markers—and perhaps their interactions with one another—influence onset and symptoms of schizophrenia.

Our finding of a positive association of levels of classical complement components C1q, C3 and C4 with thickness of the dorsolateral prefrontal cortex in healthy controls may reflect a normal function of the classical pathway in clearing away excess neurotransmitters, removing aged proteins, pruning synapses and modulating adult neurogenesis^27,59^. Higher levels of C4 have been linked to greater surface area and thickness of the insular and middle temporal cortices in healthy individuals^12^. Presumably, activation of complement pathways must be tightly regulated to prevent harmful excesses, similar to inflammatory and immune responses. Future research should elucidate the pathways that regulate complement activation and explain why they fail in schizophrenia.

### Limitations

We did not genotype the study participants, which prevents us from determining to what extent the observed differences in expression of complement components is due to one or more genetic mutations^11^. The cross-sectional study design prevents us from establishing cause-effect relationships among complement levels, cortical thinning, and logical memory. Indeed, we failed to detect significant links between cortical thinning and worse logical memory. Clearly much more work is needed into whether and how complement pathways contribute to schizophrenia, preferably through large, longitudinal studies.

## Conclusion

Our results strengthen the evidence for a role of the complement system in the pathophysiology of schizophrenia by linking hyperactivation of the classical, lectin and alternative pathways to deficits in logical memory and cortical thinning in the brain. At the same time, our work highlights many questions that remain to be resolved about the interplay of complement responses and neural processes in schizophrenia.

## Availability of data and materials

The data in this study cannot be deposited in a public repository because of privacy regulations and the conditions of the informed consent provided by study participants. Instead, data in this study are available from the corresponding author on reasonable request.

### Supplementary information

Supplementary materials

## Data Availability

The statistical maps that support the findings of this study are available from the corresponding author upon reasonable request. The data are not publicly available due to them containing information that could compromise research participant privacy or consent. Explicit consent to deposit raw-imaging data was not obtained from the patients.
