# Supplementary material for: Association of elevated levels of peripheral complement components with cortical thinning and impaired logical memory in drug-naïve patients with first-episode schizophrenia
Source: Schizophrenia (Heidelb). 2023 Nov 7;9(1):79. doi: 10.1038/s41537-023-00409-1 (PMC10630449; doi:10.1038/s41537-023-00409-1)
Supplement: Supplementary file 1 — Supplementary materials [file 41537_2023_409_MOESM1_ESM.docx]

**Supplementary materials and methods section**

**Details of the cognitive function test**

The trail-making test (TMT) provides information on visual search, scanning, speed of processing, mental flexibility, and executive functions ^1^. Trail Making Test A consists of 25 circles, distributed over a sheet of paper. Circles are numbered 1–25, and the participant is asked to draw lines to connect the numbers in ascending order as quickly as possible. Task requirements are similar for TMT-B except the person must alternate between numbers and letters (e.g., 1, A, 2, B, 3, C, etc.). The score on each part represents the amount of time required to complete the task. A lower score indicates better performance ^2^. These measurements were found to be **a** predictor of dementia in a prospective study of Swedish mild cognitive impairment patients^3^.

Digital Symbol Substitution Test (DSST) ^4^ measures working memory, visuospatial processing, attention, and processing speed. In the DSST test, nine pairs of numbers and symbols were displayed at the top of the monitor (a key). Below the key, numbers 1–9 is shown in random order. The participant is allowed 90 s to fill in the corresponding symbol for each number. Each correct pairing is scored 1 (maximum total raw score = 90). Raw scores can be converted to standard scores. A target symbol was presented at the center of the monitor. Participants were asked to choose the number corresponding to the target symbol at the bottom of the monitor. This test is a valid and reliable measure for detecting early signs of cognitive decline ^5^ and was proved to be a more sensitive measure of dementia than the **Mini-Mental State Examination score** ^6^.

The logical memory test consisted of two parts, the participants are read a logically organized story and asked to recall the story immediately after its presentation (Immediate Recall). Approximately 20 minutes later, the participants are again asked to recall the story from memory (Delayed Recall). The version used in this study uses only one story (Story A) read once to participants at each study visit. Possible scores for both Logical Memory Immediate and Delayed Recall trials range from 0 to 25, with higher scores reflecting more details recalled ^7^.

**Measurement of complement component levels**

The sensitivities of the complement components are listed as follows: Complement C1q: 0.048 ng/mL, Complement C3: 0.120 ng/mL, Complement C3b/iC3b: 3.639 ng/mL, Complement C4: 0.191 ng/mL, Complement Factor B: 0.024 ng/mL, Complement Factor H: 0.135 ng/mL and Complement Properdin 0.0032 ng/mL. The intra-assay coefficient of variation of the panel is 10%. We also provided the standard curves below in order to see these sensitivities more clearly.

*
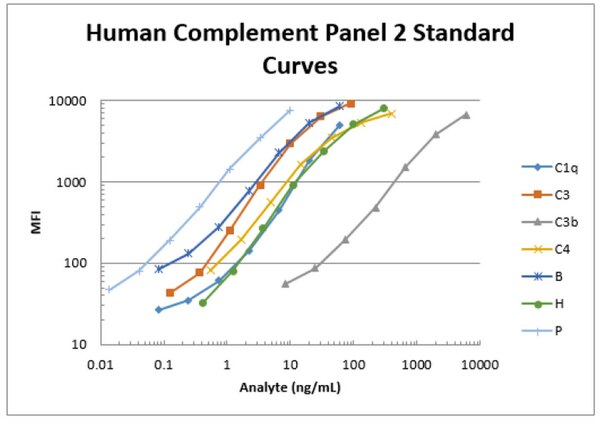
*

**Supplementary Figure 1. The standard curves of the plasma factors.**

**
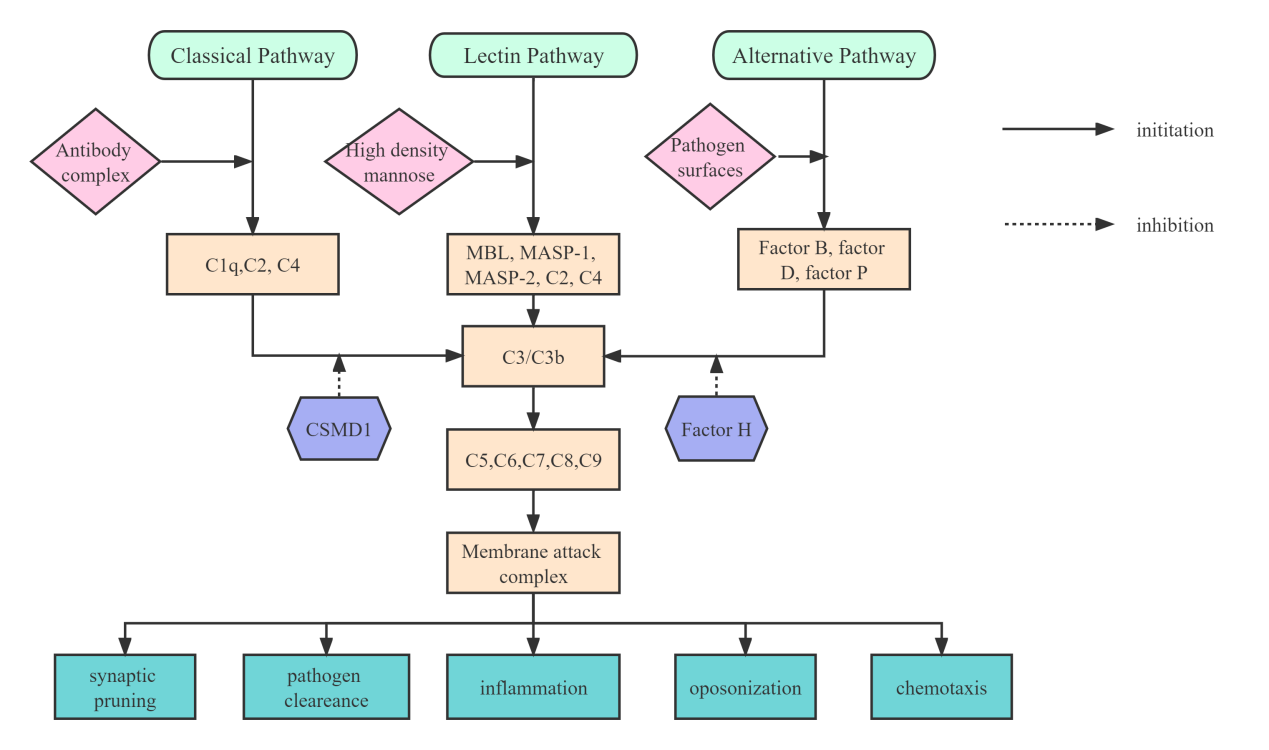
**

**Supplementary Figure 2. The three pathways of complement components.** This three pathways including the classical pathway, the lectin pathway and the alternative pathway. The classical pathway is activated by antigen-antibody reaction; the alternative pathway is activated on microbial cell surfaces, and the mannose-binding lectin pathway is activated by a plasma lectin that binds to mannose residues on microbes. All three pathways will activiate the downstream complement factors C5, C6, C7, C8 and C9, and finally generate the menbrane attack complex.

**Results**


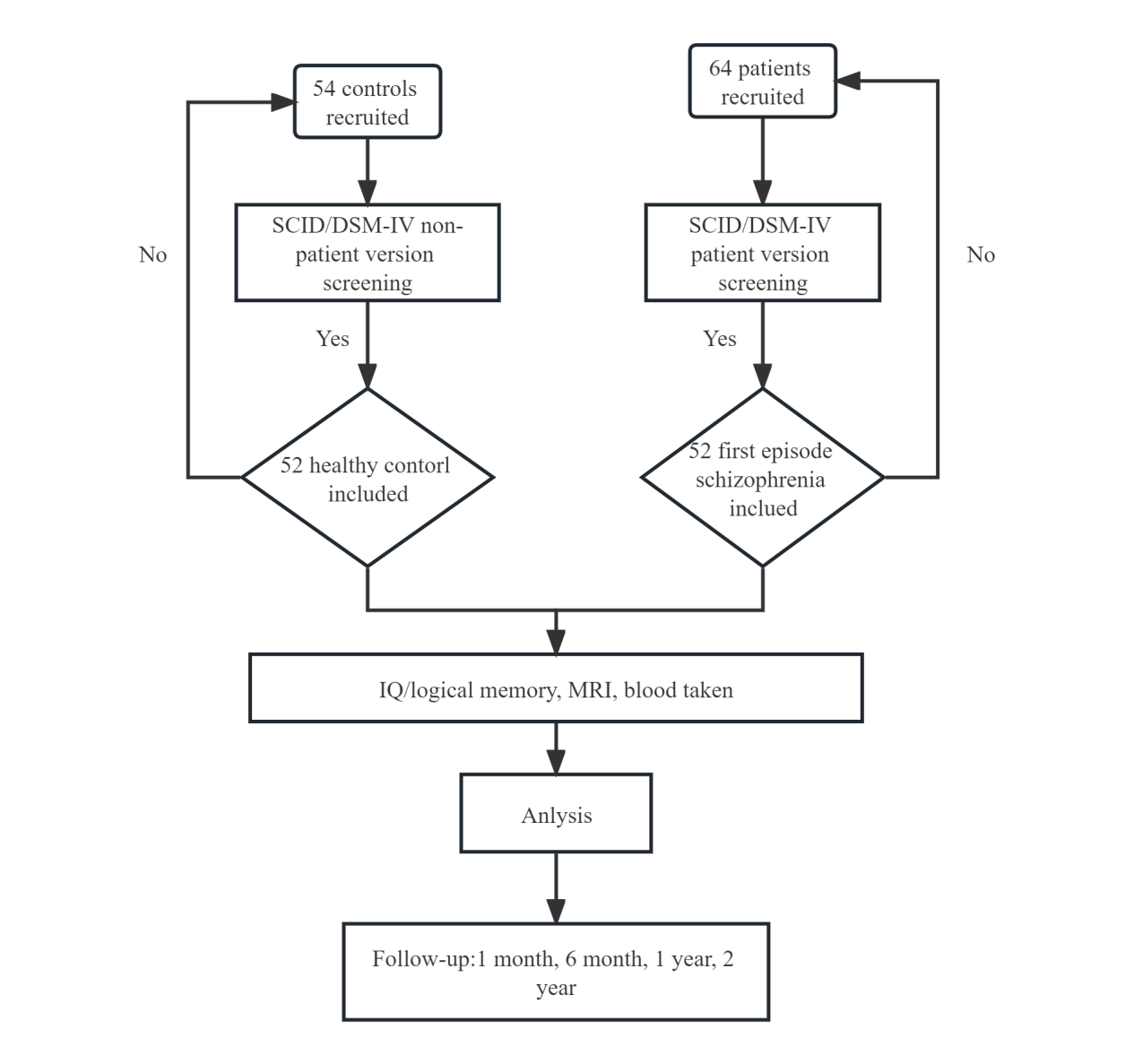
**Supplementary figure 3. The inclusion of the study participants.**

**Group comparison of cortical thickness**

Compared to the control group, the FES patients showed significantly reduced cortical thickness in the right primary motor cortex. The Montreal Neurological Institute (MNI) coordinates were: X=13/2, Y= -41/-36, Z=62/70, t = 4.30/4.28, and the cluster size was 173, with a corrected p < 0.001. This difference persisted even after the whole brain correction, controlling for age, sex, BMI, education level, and TIV. These results are illustrated in Supplementary Figure 4.


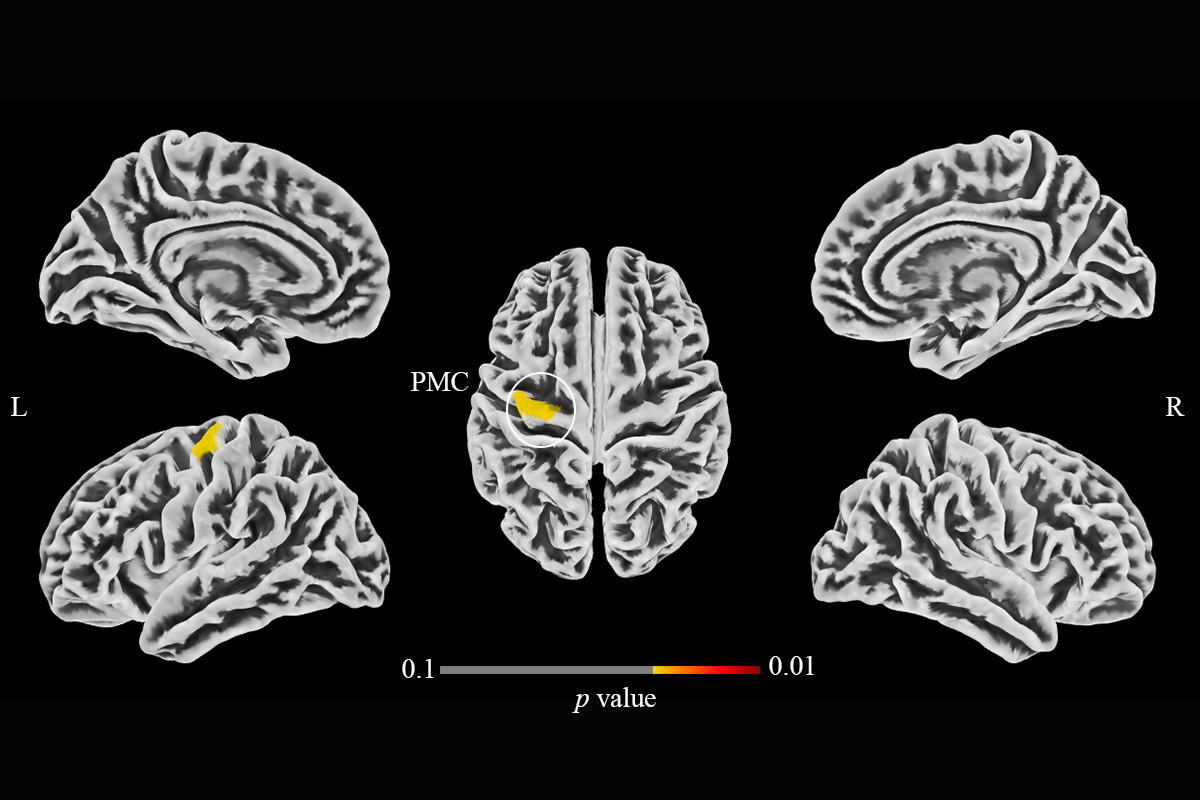


**Supplementary Figure 4．Group comparison of cortical thickness between patients and controls.**

Significant cortical thickness (CT) difference between FES and HC. Compared with HC, the FES group showed decreased CT in the right primary motor cortex (PMC) adjusted for age, sex, and education years. Blue-colored region (in white circle) shows the area with significantly lower CT in participants with FES compared with controls (cluster p < 0.05, FWE corrected; vertex level *p*_uncorrected_< 0.001).

Abbreviations: CT, cortical thickness; FES: first episode schizophrenia; PMC: primary motor cortex; FWE: family wise error.

**Discussion**

# Our finding of decreased cortical thickness in the right primary motor cortex in FES patients is of significant interest. Abnormal motor control has been implicated in schizophrenia and fist episode psychosis for a long time, suggesting a possible central role for motor dysfunctions in the pathophysiology of the psychotic spectrum disorders ^8,9^. The primary motor cortex, alongside other regions such as the supplementary motor area, dorsal anterior cingulate cortex, prefrontal cortex, basal ganglia, and cerebellum, forms part of the well-delineated neurobiological architecture of the motor system in primates ^10^. It's been suggested that the maturation of the human motor system and the emergence of motor symptoms observed in schizophrenia are closely intertwined ^10^. Supporting this, previous post-mortem and neuroimaging studies have reported structural and functional anomalies in the premotor and motor cortices, basal ganglia, thalamus, and their connecting white matter tracts in schizophrenia patients ^10^. Our findings align with these previous reports, highlighting the reduced cortical thickness of the primary motor cortex in schizophrenia subjects ^11^. This observation could contribute to a better understanding of motor dysfunction in schizophrenia. Further investigation into this area is warranted

**Supplementary Table 1. Pearson correlation between different complement factors among 104 subjects.**

|  | Log_10_ C1q (r/p) | Log_10_ C3 | Log_10_ C4 | Log_10_ Factor B | Log_10_ Factor H | Log_10_ Properdin |
| --- | --- | --- | --- | --- | --- | --- |
| Log_10_ C1q | 1.00 | 0.68 (0.00)^***^ | 0.81 (0.00)^***^ | 0.87 (0.00)^***^ | 0.88 (0.00)^***^ | 0.92 (0.00)^***^ |
| Log_10_ C3 | 0.68 (0.00)^***^ | 1 | 0.76 (0.00)^***^ | 0.72 (0.00)^***^ | 0.78 (0.00)^**^ | 0.70 (0.00)^***^ |
| Log_10_ C4 | 0.81 (0.00)^***^ | 0.76 (0.00)^***^ | 1 | 0.91 (0.00)^***^ | 0.92 (0.00)^***^ | 0.85 (0.00)^***^ |
| Log_10_ Factor B | 0.87 (0.00)^***^ | 0.72 (0.00)^***^ | 0.91 (0.00)^***^ | 1 | 0.94 (0.00)^***^ | 0.87 (0.00)^***^ |
| Log_10_ Factor H | 0.88 (0.00)^***^ | 0.78 (0.00)^***^ | 0.92 (0.00)^***^ | 0.94 (0.00)^***^ | 1 | 0.94 (0.00)^***^ |
| Log_10_ Properdin | 0.92 (0.00)^***^ | 0.70 (0.00)^***^ | 0.85 (0.00)^***^ | 0.87 (0.00)^***^ | 0.94 (0.00)^***^ | 1 |

1. Pearson correlation was conducted within complement factors.
2. Abbreviations: r, correlation coefficient; *p<0.05, **p<0.01, ***p<0.001.

# Supplementary Table 2. Association between the complement component and cognitive function in the combined group including controls and FES patients

|  | Log_10_ C1q (r/p) | Log_10_ C3 (r/p) | Log_10_ C4 (r/p) | Log_10_ Factor B (r/p) | Log_10_ Factor H (r/p) | Log_10_ Properdin (r/p) |
| --- | --- | --- | --- | --- | --- | --- |
| FES and HC |  |  |  |  |  |  |
| Immediately logical memory recall | -0.12 (0.28) | -0.12 (0.26) | -0.22 (0.038*) | -0.18 (0.090) | -0.22 (0.035*) | -0.16 (0.12) |
| Delayed logical memory recall | -0.079 (0.46) | -0.13 (0.23) | -0.19 (0.075) | -0.14 (0.20) | -0.16 (0.14) | -0.11 (0.32) |
| FES |  |  |  |  |  |  |
| Immediately logical memory recall | -0.031 (0.85) | **-0.32 (0.043*)** | -0.28 (0.083) | -0.19 (0.25) | -0.24 (0.14) | -0.12 (0.45) |
| Delayed logical memory recall | -0.038 (0.82) | **-0.36 (0.025*)** | -0.31 (0.054) | -0.21 (0.20) | -0.26 (0.11) | -0.11 (0.52) |
| HC |  |  |  |  |  |  |
| Immediately logical memory recall | 0.011 (0.94) | 0.15 (0.33) | 0.0060 (0.97) | 0.0080 (0.96) | 0.0010 (0.99) | -0.028 (0.85) |
| Delayed logical memory recall | 0.028 (0.85) | 0.11 (0.48) | 0.033 (0.82) | 0.057 (0.71) | 0.096 (0.52) | 0.016 (0.91) |

1. Partial correlation was conducted between complement factors and cognitive measurement with age, gender, education years and BMI as covariates.
2. Abbreviation: TMT: trial making test; DSST: digital symbol substitution test.
3. R, partial correlation coefficient; *p<0.05, **p<0.01, ***p<0.001. Bold represents statistically significant results (p<0.05).

**Supplementary Table 3a. Partial correlation analysis between clinical parameters and complement components in FES group**

| **Clinical Parameters** | **Log_10_ C1q (r/p)** | **Log_10_ C3 (r/p)** | **Log_10_ C4 (r/p)** | **Log_10_ Factor B (r/p)** | **Log_10_ Factor H (r/p)** | **Log_10_ Properdin (r/p)** |
| --- | --- | --- | --- | --- | --- | --- |
| **PANSS total score** | -0.045 (0.78) | -0.024 (0.88) | -0.081 (0.61) | -0.13 (0.40) | -0.036 (0.82) | -0.032 (0.84) |
| **PANSS positive scale** | 0.011 (0.95) | 0.054 (0.74) | -0.087 (0.58) | -0.095 (0.55) | -0.040 (0.80) | -0.034 (0.83) |
| **PANSS negative scale** | -0.10 (0.52) | -0.091 (0.57) | -0.10 (0.51) | -0.13 (0.41) | -0.084 (0.60) | -0.082 (0.61) |
| **PANSS general psychopathology scale** | -0.0090 (0.96) | 0.0010 (0.997) | -0.025 (0.87) | -0.098 (0.54) | 0.019 (0.90) | 0.023 (0.89) |
| **Illness duration (month)** | -0.016 (0.93) | -0.13 (0.46) | -0.14 (0.44) | -0.11 (0.51) | -0.13 (0.47) | -0.026 (0.88) |

1. Age, sex, and body mass index were controlled.

2. Abbreviation: PANSS, the Positive and Negative Symptom Scale; FES, first episode schizophrenia.

3. *p<0.05, **p<0.01, ***p<0.001. Bold represents statistically significant results.

**Supplementary Table 3b. Partial correlation analysis between PANSS subitems and complement components in FES group**

| **PANSS sub-items** | **Log_10_ C1q (r/p)** | **Log_10_ C3 (r/p)** | **Log_10_ C4 (r/p)** | **Log_10_ Factor B (r/p)** | **Log_10_ Factor H (r/p)** | **Log_10_ Properdin (r/p)** |
| --- | --- | --- | --- | --- | --- | --- |
| **P1** | 0.00 (0.99) | -0.12 (0.46) | -0.15 (0.35) | -0.16 (0.30) | -0.11 (0.48) | -0.12 (0.46) |
| **P2** | 0.16 (0.31) | 0.12 (0.47) | 0.055 (0.73) | 0.12 (0.45) | 0.087 (0.59) | 0.10 (0.51) |
| **P3** | -0.15 (0.34) | -0.058 (0.71) | -0.17 (0.29) | -0.16 (0.31) | -0.12 (0.46) | -0.089 (0.58) |
| **P4** | -0.094 (0.56) | 0.13 (0.40) | -0.10 (0.52) | -0.14 (0.38) | -0.060 (0.71) | -0.054 (0.74) |
| **P5** | 0.20 (0.21) | 0.22 (0.16) | 0.18 (0.26) | 0.097 (0.54) | 0.16 (0.31) | 0.20 (0.22) |
| **P6** | 0.10 (0.50) | -0.058 (0.72) | -0.046 (0.77) | -0.018 (0.91) | -0.0020 (0.99) | -0.014 (0.93) |
| **P7** | -0.11 (0.48) | -0.0020 (0.99) | -0.020 (0.90) | -0.043 (0.79) | -0.048 (0.76) | -0.086 (0.59) |
| **N1** | -0.053 (0.74) | -0.17 (0.29) | -0.16 (0.32) | -0.13 (0.42) | -0.12 (0.46) | -0.13 (0.43) |
| **N2** | -0.18 (0.25) | -0.21 (0.18) | -0.26 (0.094) | -0.24 (0.12) | -0.24 (0.13) | -0.24 (0.13) |
| **N3** | -0.14 (0.38) | -0.10 (0.52) | -0.17 (0.27) | -0.16 (0.31) | -0.13 (0.40) | -0.13 (0.40) |
| **N4** | -0.057 (0.72) | -0.086 (0.59) | -0.11 (0.48) | -0.11 (0.48) | -0.076 (0.63) | -0.010 (0.95) |
| **N5** | 0.0030 (0.98) | 0.076 (0.63) | 0.11 (0.50) | 0.013 (0.93) | 0.068 (0.67) | 0.028 (0.86) |
| **N6** | -0.10 () | -0.084 (0.60) | -0.048 (0.76) | -0.14 (0.39) | -0.041 (0.80) | -0.028 (0.86) |
| **N7** | -0.062 (0.69) | 0.040 (0.80) | 0.024 (0.88) | 0.0030 (0.99) | 0.035 (0.83) | 0.017 (0.91) |
| **G1** | 0.027 (0.86) | -0.12 (0.45) | -0.059 (0.71) | -0.078 (0.62) | -0.046 (0.77) | 0.079 (0.62) |
| **G2** | 0.10 (0.52) | 0.11 (0.47) | 0.18 (0.25) | 0.092 (0.56) | 0.20 (0.21) | 0.12 (0.46) |
| **G3** | 0.11 (0.49) | -0.0010 (0.99) | 0.13 (0.40) | 0.080 (0.62) | 0.14 (0.38) | 0.087 (0.58) |
| **G4** | 0.13 (0.43) | -0.22 (0.17) | -0.074 (0.64) | 0.000 (1.00) | -0.031 (0.85) | 0.014 (0.93) |
| **G5** | 0.19 (0.22) | 0.27 (0.085) | 0.19 (0.24) | 0.066 (0.68) | 0.18 (0.26) | 0.25 (0.11) |
| **G6** | -0.055 (0.73) | -0.022 (0.89) | -0.029 (0.86) | -0.014 (0.93) | -0.0010 (0.99) | -0.0090 (0.95) |
| **G7** | -0.16 (0.33) | -0.081 (0.61) | -0.028 (0.86) | -0.11 (0.48) | -0.034 (0.83) | -0.063 (0.69) |
| **G8** | -0.044 (0.78) | 0.50 (0.35) | 0.013 (0.93) | -0.053 (0.74) | -0.0020 (0.99) | 0.020 (0.90) |
| **G9** | 0.016 (0.92) | -0.035 (0.83) | -0.10 (0.49) | -0.089 (0.58) | -0.030 (0.85) | -0.063 (0.69) |
| **G10** | 0.0080 (0.96) | 0.10 (0.53) | 0.071 (0.65) | -0.052 (0.74) | 0.031 (0.84) | 0.083 (0.60) |
| **G11** | 0.099 (0.53) | -0.0060 (0.97) | 0.042 (0.79) | 0.046 (0.77) | 0.065 (0.68) | 0.15 (0.34) |
| **G12** | -0.20 (0.21) | -0.058 (0.71) | -0.21 (0.17) | -0.22 (0.16) | -0.15 (0.34) | -0.18 (0.25) |
| **G13** | -0.17 (0.27) | -0.017 (0.92) | -0.034 (0.83) | -0.15 (0.34) | -0.041 (0.80) | -0.10 (0.53) |
| **G14** | 0.11 (0.46) | 0.14 (0.38) | 0.21 (0.19) | 0.14 (0.39) | 0.16 (0.31) | 0.097 (0.54) |
| **G15** | 0.026 (0.87) | 0.16 (0.30) | 0.039 (0.81) | -0.032 (0.84) | 0.11 (0.51) | 0.079 (0.62) |
| **G16** | -0.063 (0.69) | -0.17 (0.28) | -0.22 (0.17) | -0.19 (0.23) | -0.17 (0.28) | -0.12 (0.46) |
| **S1** | 0.21 (0.18) | 0.25 (0.11) | 0.23 (0.14) | 0.17 (0.28) | 0.23 (0.15) | 0.16 (0.32) |
| **S2** | 0.039 (0.80) | -0.12 ()0.46 | 0.028 (0.86) | 0.000 (1.00) | 0.011 (0.94) | 0.070 (0.66) |
| **S3** | -0.021 (0.90) | 0.12 (0.47) | -0.012 (0.94) | -0.044 (0.78) | 0.023 (0.89) | -0.0090 (0.96) |

1. Age, sex, and body mass index were controlled.

2. Abbreviation: PANSS, the Positive and Negative Symptom Scale; P: positive scale; N:negative scale; G: general pathology scale; S: supplemenatry scale; FES, first episode schizophrenia.

3. *p<0.05, **p<0.01, ***p<0.001. Bold represents statistically significant results.

**References**

1 Tombaugh, T. N. Trail Making Test A and B: normative data stratified by age and education. *Archives of clinical neuropsychology* **19**, 203-214 (2004).

2 Llinàs-Reglà, J. *et al.* The Trail Making Test. *Assessment* **24**, 183-196 (2017).

3 Innes, K. E., Selfe, T. K., Khalsa, D. S. & Kandati, S. Meditation and Music Improve Memory and Cognitive Function in Adults with Subjective Cognitive Decline: A Pilot Randomized Controlled Trial. *Journal of Alzheimer's disease* **56**, 899-916 (2017).

4 Jaeger, J. Digit Symbol Substitution Test: The Case for Sensitivity Over Specificity in Neuropsychological Testing. *J Clin Psychopharmacol* **38**, 513-519 (2018).

5 Donohue, M. C. *et al.* The preclinical Alzheimer cognitive composite: measuring amyloid-related decline. *JAMA Neurol* **71**, 961-970 (2014).

6 Fried, L. P. *et al.* Risk factors for 5-year mortality in older adults: the Cardiovascular Health Study. *Jama* **279**, 585-592 (1998).

7 Gavett, B. E. *et al.* Practice Effects on Story Memory and List Learning Tests in the Neuropsychological Assessment of Older Adults. *PLoS One* **11**, e0164492 (2016).

8 Presta, V. *et al.* Posture and gait in the early course of schizophrenia. *PloS one* **16**, e0245661 (2021).

9 Riley, E. M. *et al.* Neuropsychological functioning in first-episode psychosis--evidence of specific deficits. *Schizophr Res* **43**, 47-55 (2000).

10 Walther, S. & Strik, W. Motor symptoms and schizophrenia. *Neuropsychobiology* **66**, 77-92 (2012).

11 Walker, E., Lewis, N., Loewy, R. & Palyo, S. Motor dysfunction and risk for schizophrenia. *Development and psychopathology* **11**, 509-523 (1999).
